# Giant Fibrolipoma of the Esophagus

**DOI:** 10.1155/2012/406167

**Published:** 2012-02-20

**Authors:** Ryan L. Kau, Alpen B. Patel, Michael L. Hinni

**Affiliations:** Department of Otolaryngology-Head and Neck Surgery, Mayo Clinic Hospital, Phoenix, AZ 85054, USA

## Abstract

Benign tumors of the esophagus are uncommon, representing <0.5% of esophageal tumors. Fibrolipomas are a subset of benign fibrovascular tumors, which present with dysphagia, odynophagia, and substernal fullness. These intraluminal tumors can become elongated and molded into a long pedunculated polyp by constant peristaltic movements. They can cause esophageal obstruction if large and long enough and can cause asphyxiation if they become lodged into the glottis. A barium swallow is the main diagnostic tool; treatment is surgical via a transoral, transcervical, or transthoracic approach. We report the excision of a large esophageal fibrolipoma through a transoral laser microsurgical approach.

## 1. Introduction

Benign tumors of the esophagus are uncommon, representing fewer than 0.5% of all esophageal tumors [1, 2]. Fibrovascular tumors which include fibrolipomas, fibromas, myomas, myxofibromas, lipomas, and fibroepithelial polyps are a subset of benign esophageal tumors. These tumors manifest mostly with dysphagia and odynophagia, as the mass of the tumors become substantial enough to compress the lumen of the esophagus, be it intramural or intraluminal. These benign tumors necessitate an open, usually transcervical, surgical approach to adequately remove the lesions. We present an unusual case of a giant fibrolipoma, which was removed transorally with the CO_2_ laser.

## 2. Case Report

**Figure 1:**
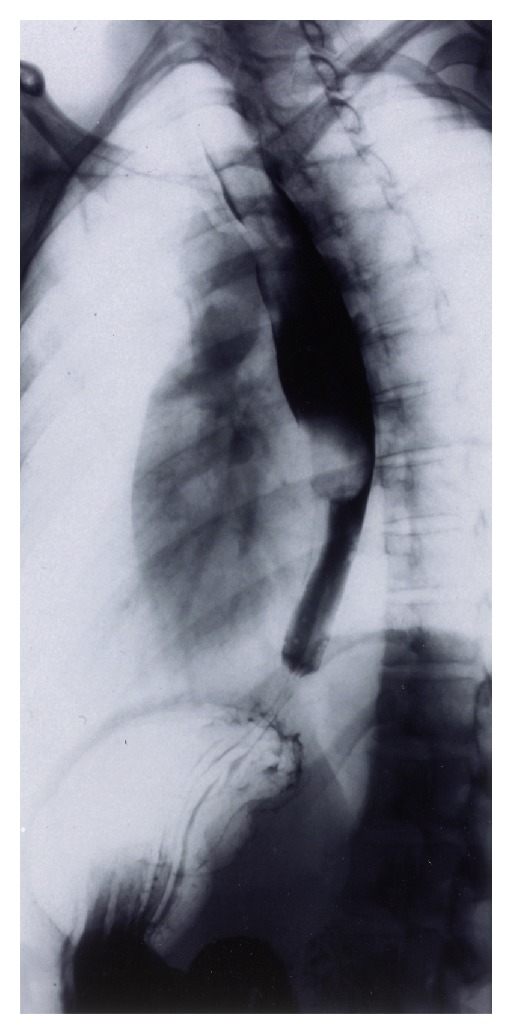
Barium esophagram showing a long pedunculated intraluminal mass from the cricopharyngeus muscle to the level of the inferior pulmonary veins. Unimpeded flow of barium to the stomach was observed.

A 38-year-old woman was referred to our institution for evaluation of a 6-month history of sore throat and left neck pain. The pain became intense enough to require narcotic pain control. Progressive odynophagia and dysphagia to solid food also developed. She had had a 4.5 kg weight loss before presentation. Physical examination of the head and neck region was normal, including fiberoptic visualization of the larynx and hypopharynx. A barium swallow study indicated a long, pedunculated intraluminal mass, originating from the upper esophagus and extending inferiorly below the level of the inferior pulmonary veins (Figure 1). It was estimated that the mass was 23 cm long and 3 cm in diameter. With the presumptive diagnosis of an intraluminal lipoma, a flexible esophagoscopy was performed; it revealed a smooth mucosa-covered, pedunculated mass arising from the posterior aspect of the esophagus, below the cricopharyngeus, with an estimated vertical dimension of 21 cm. The inferior apex of the mass appeared ulcerated. Biopsies of the ulcerated region showed reactive squamous mucosa with candidal colonization.

**Figure 2:**
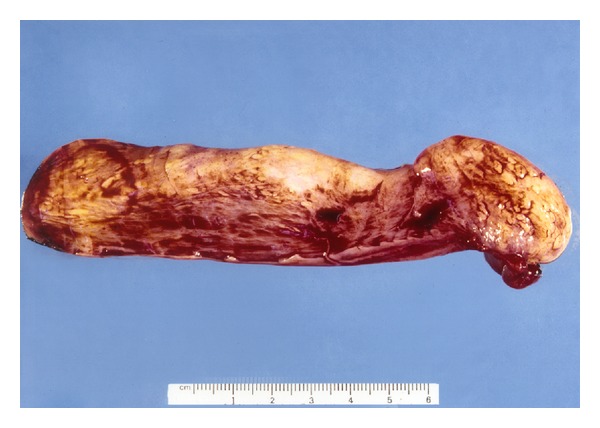
Resected fibrolipoma specimen measuring 15 × 2.6 cm and weighing 53 g. The inferior apex of the polyp (at right) shows ulceration due to reflux of gastric content.

**Figure 3:**
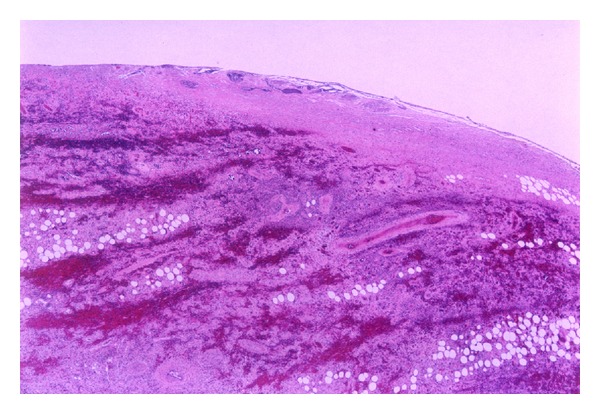
Histologic specimen of fibrolipoma on H&E stain. A variable amount of adipose tissue with a fibrovascular stroma covered by normal squamous mucosa.

Subsequently, direct laryngopharyngoscopy was performed with a Weerda laryngoscope. After adequately exposing the superior stalk of the mass, a right angle clamp was used on the right side of the stalk. The CO_2_ laser was then used in conjunction with the operating microscope to make an incision through the right side of the stalk. This was then repeated on the left, resulting in detachment of the polyp. A bipolar was used to control any bleeding. The lesion, which was retrieved through the Weerda laryngoscope, measured 15 cm × 2.6 cm and weighed 53 g (Figure 2). Histologic examination of the mass revealed variable amounts of adipose tissue and stroma were consistent with a fibrolipoma (Figure 3). Reexamination of the esophagus showed no esophageal perforation, and a feeding tube was placed under direct visualization. The patient was placed on a clear liquid diet on the second postoperative day. At the 2-week follow-up visit, she had near-complete resolution of the dysphagia and odynophagia and was tolerating a regular diet. The patient had an unremarkable postoperative course and has had no further recurrences during the past 10 years.

## 3. Discussion

Benign tumors of the esophagus are rare; the exact incidence is unknown because many are discovered postmortem [3]. Moersch and Harrington found 44 benign esophageal tumors in 7,459 consecutive autopsies at the Mayo Clinic, for a prevalence of 0.59% [1].

Plachta reported 90 benign esophageal tumors from 19,982 consecutive autopsies over a 50-year period: a 0.45% prevelance [2]. The vast majority of these benign tumors are intramural of muscular tissue origin, representing leiomyomas. In a Mayo Clinic study of 246 benign esophageal tumors, 145 (59%) were leiomyomas, 55 (22%) were cysts, and only 12 (5%) were intrauminal polyps [3, 4]. Intramural lesions typically consist of leiomyomas, neurofibromas, and hemangiomas, whereas intraluminal lesions include fibrovascular polyps, hamartomas, lymph myomas, and lipomas [5].

Histologically, fibrovascular tumors are composed of variable amounts of adipose tissue and stroma covered by normal squamous mucosa [3]. The male-to-female ratio is 3 : 1, and the tumors are usually discovered during the sixth or seventh decade of life [5, 6]. This type of tumor is believed to begin as a nodular submucosal thickening or fold of redundant mucosa in a narrow segment of the esophagus. The lesions is slowly elongated and molded by continuous peristalsis into a pedunculated polyp [5, 6].

Symptoms from fibrovascular esophageal polyps include dysphagia, odynophagia, substernal fullness, gastroesophageal reflux, fever, melena, hiccups, and the regurgitation of undigested food [3, 5]. Weight loss becomes a more prominent symptom as the tumor increases in size [3]. Obstruction can occur if the tip of the lesion becomes impacted at the gastroesophageal junction. Ulceration at the inferior apex of the lesion can occur from gastric acid and contents refluxing into the distal esophagus (as was seen in this case) and can lead to occult bleeding [3]. Partial regurgitation of a fleshy mass into the mouth with disappearance upon swallowing can be a dramatic presentation [3, 7]. Asphyxiation from impaction of the polyp in the glottis is the most feared complication of these benign tumors [3, 8]. Malignant degeneration of fibrovascular polyps is extremely rare. In a report by Kuwano et al. on the pathologic findings of 587 patients with esophageal cancer treated surgically, only 3 cases were associated with benign tumors, 2 were leiomyomas, and 1 was a lipoma [9].

The diagnosis of a fibrovascular polyp is often difficult unless actual regurgitation of the lesion occur. Unrecognized fibrovascular tumors can impact and obstruct the airway, with an associated mortality rate of up to 30% [10]. Flexible fiberoptic laryngoscopy can fail to spot the polyp due to its location and attachment in the esophagus. In fact, the polyp may be completely missed at esophagoscopy, because the polyp is covered with normal mucosa and is easily displaced by the endoscope. Barium swallow studies usually reveal a dilated hypertrophic esophagus similar to that in achalasia, or may show a smooth polypoid, intraluminal filling defect, perhaps with a stalk just inferior to the cricopharyngeus muscle (Figure 1) [3]. Fluoroscopy generally shows unimpeded contrast flow, as well as the up and down movements of the tumor during swallowing. Computed tomography and magnetic resonance imaging are probably unnecessary studies if fluoroscopy itself can demonstrate the pedunculated nature of the polyp. Esophageal ultrasonography may indicate feeding vessels in the stalk of the polyp [5].

Treatment of fibrovascular polyps is surgical. Generally, large polyps are removed through a cervical or transthoracic approach. Smaller polyps and those that attach at the level of the cricopharyngeus muscle may be amenable to transoral procedures. Transoral approaches have included an endoscopic removal with combination of bipolar and snare [11, 12], and suture ligation and snare [13]. To our knowledge, this is the first report of a giant fibrolipoma polyp removed transorally with the CO_2_ laser. CO_2_ laser has the advantages of better precision and a decreased amount of surrounding tissue damage versus electrocautery. This is due to the absorption of energy at the targeted tissue and the steep decrease in power density of the laser beyond the target. As in our case, an operating microscope further allows for magnification, binocular vision, precise laser control, and usage of one hand to control the polyp while the other controls the laser [14]. Whether the approach is transoral, transcervical, or transthoracic, the feeding vessels in the stalk should be controlled to ensure hemostasis and eliminate the possibility of recurrence [3].

In summary, fibrolipomas of the esophagus are extremely uncommon but should be part of the differential diagnosis in patients with dysphagia, weight loss, odynophagia, and substernal fullness. These tumors are difficult to diagnose, but a barium swallow is generally the key diagnostic study. Once the tumor is identified, the surgical approach needs to be tailored according to the size of the lesion, proximity of the stalk to the cricopharyngeus muscle, and the diameter of the stalk. Finally, the diagnosis of fibrolipoma requires a high index of suspicion because sudden asphyxiation due to upper airway obstruction is a tragic complication which can be avoided with the proper evaluation.
